# Collectivism reduces objective mobility trends to public areas during the COVID-19 pandemic

**DOI:** 10.3389/fpubh.2022.996036

**Published:** 2022-09-28

**Authors:** Junhua Dang, Shanshan Xiao

**Affiliations:** ^1^School of Psychology, Beijing Sport University, Beijing, China; ^2^Department of Surgical Sciences, Uppsala University, Uppsala, Sweden; ^3^Department of Psychology, Stockholm University, Stockholm, Sweden

**Keywords:** COVID-19, culture, collectivism, mobility, prevention

## Abstract

In order to slow down the spread of the coronavirus, staying at home and avoiding going outside have been either strongly recommended or stringently enforced by governments all over the globe. Previous studies found that people with more collectivist orientation were more willing to comply with governmental guidelines and engage in preventive behaviors such as social distancing. However, these studies were based on self-report data within a short period. The current study aims to overcome these limitations by using objective mobility data generated by Google users all over the world during the past two years, thus providing a stronger test for the predictive effect of collectivism on preventive measures in response to the pandemic. We found consistent results at both the US state level (*n* = 50) and the country/territory level (*n* = 133), such that people in more collectivistic regions reduced their visits to and length of stay at certain public areas such as parks during the past two years. Our findings emphasize the importance of cultural values in face of global crises.

## Introduction

The worldwide outbreak of the coronavirus disease 2019 (COVID-19), caused by the novel severe acute respiratory syndrome coronavirus 2 (SARS-CoV-2), has transpired as the most severe human crisis in recent history. As of 30 June 2022, 553 million infected cases and over 6 million deaths have been reported across 188 countries and territories (1). The virus spreads mostly through droplets that people send out when they talk, sneeze, or cough. Therefore, in order to slow down the spread of the virus, staying at home and avoiding going outside have been either strongly recommended or stringently enforced all over the globe. As the Centers for Disease Control and Prevention (CDC) suggested, “the best way to prevent illness is to avoid being exposed to this virus.”

However, people differ strikingly in their responses to recommendations and policies restricting mobility. Those in some regions like Singapore and South Korea obeyed with few complaints whereas those in many other regions like the Netherlands and the United Kingdom protested vehemently. What explains these prominent differences? We propose that regional culture is one of the most important factors.

There are many dimensions along which cultural values can be analyzed (2). Among them, collectivism-individualism is most relevant to mobility restrictions because such restrictions bring great inconvenience and thus induce a conflict between private rights and public rights. Collectivism-individualism indicates the degree to which people in a society behave in line with obligations and expectation of others, roles, and situations versus their own preferences (3). Collectivists are more concerned with the group's needs, goals, and interests whereas individualists are more concerned with their own needs, goals, and interests. In face of the pandemic and the requirements of staying at home and avoiding going outside, people in collectivist cultures may be more willing to sacrifice their personal convenience to comply with governmental guidelines that can lead to lower prevalence rate for the whole group, compared with those in individualistic cultures.

Lately, the important role of collectivism during the current pandemic has been emphasized by many researchers. For example, collectivist cultures led to slower increase in prevalence rate and mortality rate within a 30-day timeframe after a region's lockdown (4). Over a longer period, such as the first three months from the first reported case or the whole year of 2020, a positive correlation between regional collectivism and COVID-19 prevalence and mortality has also been consistently found, showing that less cases of infection and death were reported in more collectivist regions (5–8). Such regional differences may partly result from people's guideline compliance, such that people with more collectivist orientation were more willing to comply with governmental guidelines and engage in preventive behaviors such as mask wearing and social distancing (5, 6, 9–11). However, these studies were based on self-report data within a short period. The current study aims to overcome these limitations by using objective mobility data generated by Google users all over the world during the past two years, thus providing a stronger test for the predictive effect of collectivism on preventive measures in response to the pandemic.

Specifically, we test the hypothesis that people in collectivist regions are more likely to comply with governmental guidelines restricting mobility by using objective mobility data from the Google COVID-19 Community Mobility Reports (12). We aim to show how people in regions with different indices of collectivism changed their mobility trends to each place category during the past two years (from 1 March 2020 to 31 December 2021). The level of collectivism varies not only in different countries/territories but also in subregions within a specific country. Therefore, we examine the relationship between collectivism and mobility changing trends both within the United States (i.e., among the 50 US states) and across different countries/territories. Consistent results from different analytical levels would provide stronger support for our hypothesis.

## Methods

### Outcome measure

The Google COVID-19 Community Mobility Reports is a publicly accessible database (12). Based on the data of Google users who opted-in to Location History, these reports generate anonymized metrics to show what percentage of people's visits to and length of stay at different places have changed compared with a baseline in each geographic region (13). The baseline is the median value during the 5-week period from January 3 to February 6, 2020. There are six place categories: Retail and Recreation, Grocery and Pharmacy, Parks, Transit Station, Workplaces, and Residential. We computed the mean of percentage change across six categories of places for each US state and each country/territory between 1 March 2020 and 31 December 2021, which served as the outcome measure.

### US state level predictors

Vandello and Cohen's collectivism-individualism index was used in the current study, with higher scores indicating more collectivist states (14). We also controlled for a series of variables to test the robustness of the relationship between the US state level index of collectivism and mobility changing trends to each place category. These control variables included median age of each state, male to female ratio, population density, educational level (the percentage of adults with a bachelor's degree or higher in each state), income per capita, political affiliation (the percentage of adults who identify as Republican/leaning Republican), and government stringency.

Population median age, male to female ratio, population density (log-transformed due to skewness), and educational level (the percentage of adults with a bachelor's degree or higher) of each US state in 2019 were sourced from the US Census Bureau. Income per capita of each state in 2019 was sourced from the US Bureau of Economic Analysis. The percentage of adults who identify as Republican/leaning Republican in each state was sourced from the Pew Research Center (15). Finally, the government stringency index was sourced from the Oxford COVID-19 Government Response Tracker (16). We computed the mean score of the government stringency index between 1 March 2020 and 31 December 2021 for each state.

### Country/territory level predictors

At the country/territory level, the Google COVID-19 Community Mobility Reports include data from 135 countries/territories. Recent research introduced the Global Collectivism Index for 188 countries/territories (17) and this index is available for 133 of the 135 countries/territories included in the Google COVID-19 Community Mobility Reports. We also controlled for a series of variables to test the robustness of the relationship between the Global Collectivism Index and mobility changing trends to each place category. These control variables included median age of each country/territory, male to female ratio, population density, GDP per capita, the government stringency index, and the Universal Health Coverage Index from the World Health Organization.

Population median age, male to female ratio (log-transformed due to skewness), and population density (log-transformed due to skewness) of each country/territory were sourced from the United Nations' Department of Economic and Social Affairs (18). GDP per capita of each country/territory was sourced from Our World in Data (19), which was log-transformed due to skewness. The government stringency index was sourced from the Oxford COVID-19 Government Response Tracker (16). We computed the mean score of the government stringency index between 1 March 2020 and 31 December 2021 for each country/territory. The University Health Coverage Index, which measures the coverage of important health services in a country/territory, was sourced from the World Health Organization (20).

### Data analysis

First, we tested the normality of each predictor based on the skewness index. If the skewness is smaller than −1 or bigger than 1, the log-transformation was conducted, as described above. Second, we calculated the Person bivariate correlation between the collectivism index and mobility changing trends to each place category. Third, we conducted hierarchical regression to control the effect of potential confounders. In step 1, we entered all the control variables. In step 2, we entered the collectivism index to test if the collectivism index could still predict mobility changing trends above and beyond all the control variables. The significance level was determined at *p* = 0.05.

## Results

### US state level

We first test the bivariate correlation between the US state level index of collectivism and mobility changing trends to each place category. Collectivism is negatively correlated with mobility changing trends to places of Retail & Recreation, *r* = −0.470, *p* = 0.001, Grocery & Pharmacy, *r* = −0.486, *p* < 0.001, Parks, *r* = −0.593, *p* < 0.001, Transit Station, *r* = −0.337, *p* = 0.017, and Workplaces, *r* = −0.321, *p* =0.023, but positively correlated with mobility changing trends to places of Residential, *r* = 0.363, *p* = 0.010.

**Figure 1:**
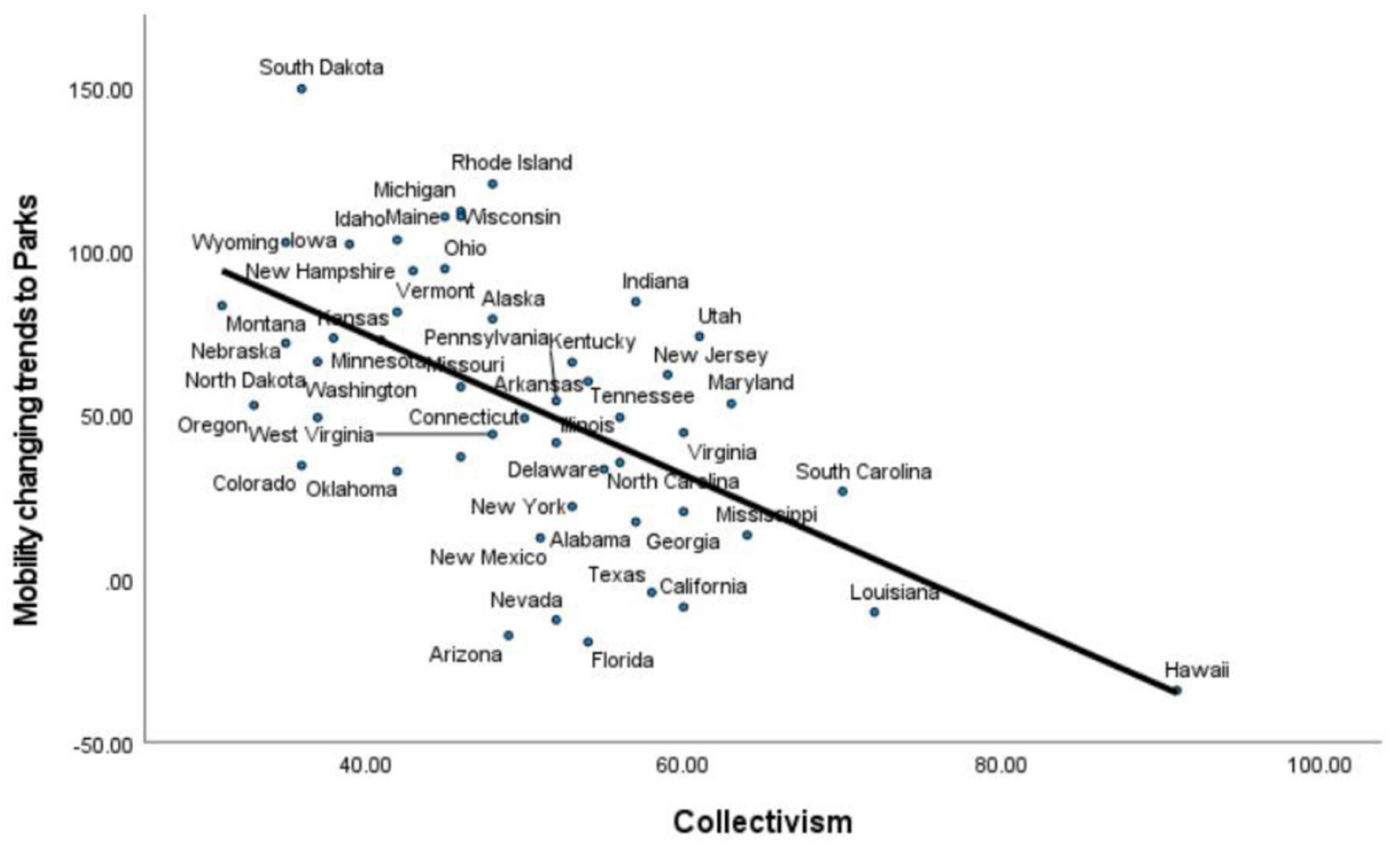
Mobility trends to Parks reduced more in more collectivist US states. Mobility changing tends to Parks means the percentage of people's visits to and length of stay at places of Parks that have changed compared with a baseline before the pandemic. Positive values denote increased visits whereas negative values denote decreased visits.

When the control variables are considered in the hierarchical regression, collectivism remains significant for places of Parks, Δ*R*^2^ = 0.139, β = −0.527, *p* = 0.003, and Residential, Δ*R*^2^ = 0.02, β = 0.200, *p* = 0.019, as shown in Table 1. The scatter plot of the relationship between the US state level index of collectivism and mobility changing trends to Parks is displayed in Figure 1.

**Table 1:** Hierarchical regression results (standardized coefficients) of mobility changing trends on control variables and collectivism at the US state level (*n* = 50).

	**Retail and recreation**	**Grocery and pharmacy**	**Parks**	**Transit stations**	**Workplaces**	**Residential**
Mean (SD)	−10.05 (6.96)	0.63 (6.23)	52.90 (41.10)	−15.07 (17.55)	−29.07 (4.54)	9.04 (2.25)
**Step 1**						
Median age	0.256^*^	0.303^*^	0.395^*^	0.174^*^	0.311^**^	−0.231^**^
Male to female ratio	−0.378	−0.342	0.157	−0.376^**^	−0.184	0.281^*^
% Bachelor degree or above	−0.048	0.093	0.301	−0.301^**^	−0.340^*^	0.243^*^
Population density (log)	−0.774^**^	−0.750^**^	−0.128	−0.630^***^	−0.462^*^	0.511^**^
Income per capita	0.154	0.074	0.086	0.184	0.211	−0.032
% Republican	0.279	0.344	0.414	0.541^***^	0.512^***^	−0.528^***^
Government stringency	−0.324^**^	−0.241	−0.152	−0.058	−0.141	0.085
Unique *R*^2^	0.681^***^	0.615^***^	0.285^*^	0.848^***^	0.782^***^	0.842^***^
**Step 2**						
Collectivism	−0.141	−0.169	−0.527^**^	−0.103	−0.032	0.200^*^
Unique ***R*^2^**	0.010	0.014	0.139^**^	0.005	0.001	0.020^*^

* *p* < 0.05,

** *p* < 0.01,

*** *p* < 0.001 (2-tailed).

### Country/territory level

**Figure 2:**
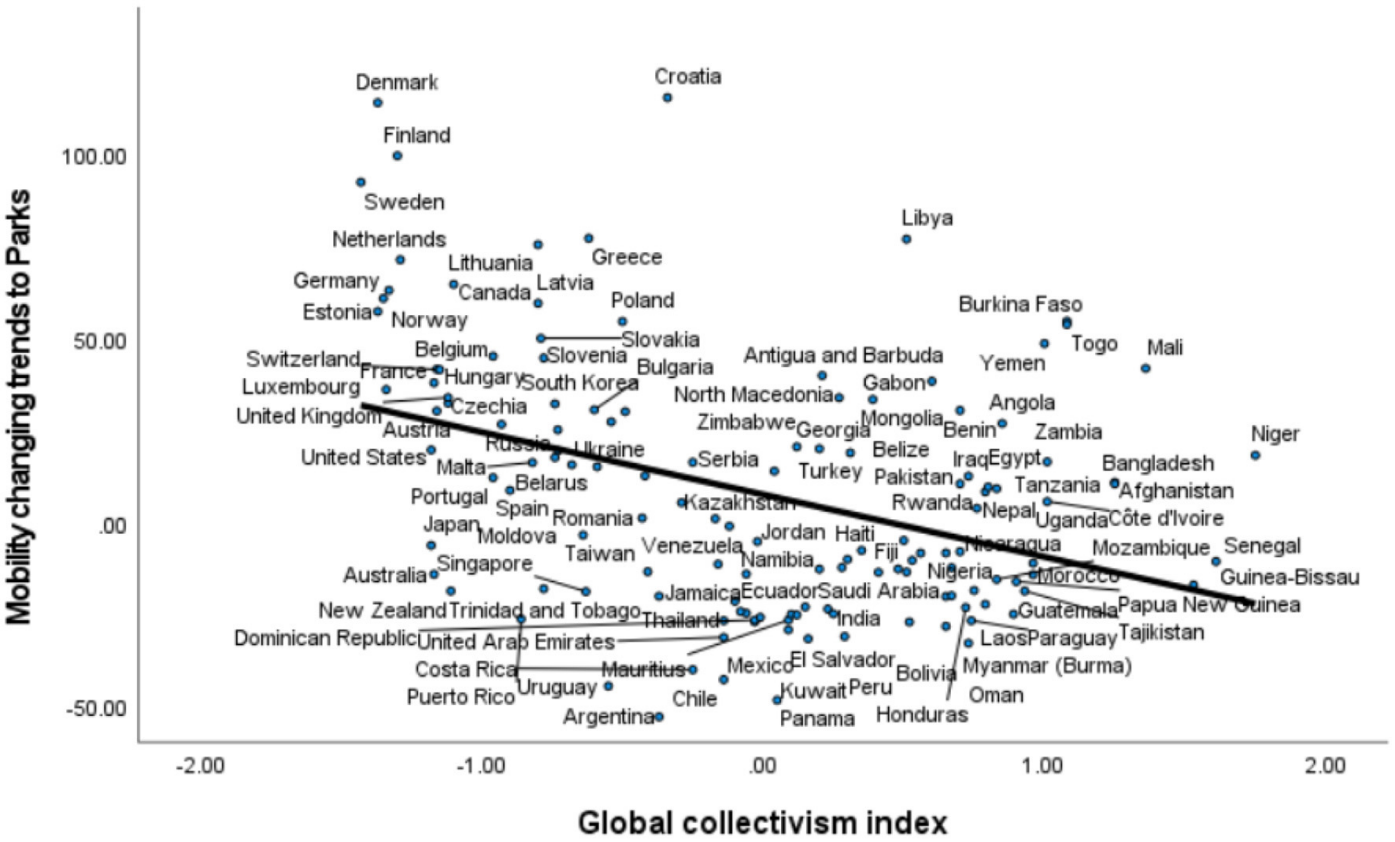
Mobility trends to Parks reduced more in more collectivist countries/territories.

At the country/territory level, the Global Collectivism Index is negatively correlated with mobility changing trends to places of Parks, *r* = −0.383, *p* < 0.001, which remains significant when the control variables are considered, Δ*R*^2^ = 0.045, β = −0.524, *p* = 0.005, as shown in Table 2. Although collectivism is positively correlated with mobility changing trends to places of Retail and Recreation, *r* = 0.346, *p* < 0.001, Grocery and Pharmacy, *r* = 0.284, *p* = 0.001, Transit Station, *r* = 0.359, *p* < 0.001, and Workplaces, *r* = 0.398, *p* < 0.001, all these relationships become non-significant when the control variables are considered. The scatter plot of the relationship between the Global Collectivism Index and mobility changing trends to Parks is displayed in Figure 2.

**Table 2:** Hierarchical regression results (standardized coefficients) of mobility changing trends on control variables and collectivism at the country/territory level (*n* = 123).

	**Retail and recreation**	**Grocery and pharmacy**	**Parks**	**Transit stations**	**Workplaces**	**Residential**
Mean (SD)	−13.79 (15.88)	7.74 (18.95)	7.86 (34.53)	−19.10 (18.52)	−18.19 (9.49)	6.69 (4.96)
**Step 1**						
Median age	0.083	−0.022	0.582^**^	0.055	0.038	−0.741^***^
Male to female ratio (log)	0.038	−0.078	−0.216^*^	−0.038	0.128	−0.129
Population density (log)	−0.227^**^	−0.165	−0.143	−0.105	−0.194^*^	0.358^***^
GDP per capita (log)	0.062	0.297	0.290	0.086	−0.087	0.348
Universal health coverage	−0.635^***^	−0.648^***^	−0.527^**^	−0.611^***^	−0.477^**^	0.479^**^
Government stringency	−0.321^***^	−0.134	−0.221^**^	−0.220^**^	−0.200^**^	0.315^***^
Unique *R*^2^	0.523^***^	0.284^***^	0.318^***^	0.369^***^	0.414^***^	0.410^***^
**Step 2**						
Collectivism	−0.239	−0.008	−0.524^**^	−0.244	−0.111	0.099
Unique *R*^2^	0.009	0.000	0.045^**^	0.010	0.002	0.002

* p < 0.05,

** *p* < 0.01,

*** *p* < 0.001 (2-tailed).

## Discussion

In summary, our results at the US state level and the country/territory level are consistent, showing that collectivism is a robust predictor of mobility changing trends to certain public areas such as parks. That is to say, compared with the time before the pandemic, people in collectivistic cultures have reduced their visits to and length of stay at the place category of Parks, which may help to slow down the spread of the virus. In addition, as can be seen in the second row of both Tables 1, 2, people generally reduced mobility to public areas except Parks. For example, in the US, residents in almost all states increased their mobility to Parks, which was weakened by regional collectivism. Therefore, policy makers may need to pay special attention to the place category of Parks, at which other preventive measures should be enhanced.

In the literature, many researchers adopted Hofstede's index of individualism/collectivism at the country/territory level. However, this index has been criticized for several reasons. First, it has limited global coverage that is strongly biased toward WEIRD (Western, Educated, Industrialized, Rich, and Democratic) regions. Second, this index was based primarily on IBM employees rather than representative samples. Third, this index was based mainly on data collected half century ago. In the current study, we adopted the very recent Global Collectivism Index (17). This new index was available for 188 countries/territories, thus having a very high global coverage. Further, it was based on six representative indicators of collectivism: total fertility rate, living arrangements, religiosity, collective transportation, interdependent attitudes, and entrepreneurship. All these indicators were based on data after 2009. Due to these advantages (updated data and high global coverage resulting in a large sample size for analysis), the Global Collectivism Index should be preferred. Meanwhile, in addition to individualism/collectivism, Hofstede also proposed other cultural dimensions such as uncertainty avoidance and power distance. Based on the same rationale, however, the indices of these dimensions also tend to result in biased estimates for the predictive effects of culture. Therefore, more updated indices like the Global Collectivism Index need to be developed for other cultural dimensions. Further, there are no measures available for other cultural dimensions at the US state level. Therefore, we also call for such indices in future research.

There are several limitations in the current study. First, we used Vandello and Cohen's collectivism-individualism index (14) to measure the US state level collectivism, which is the only scale currently available and has been extensively used. Although it is younger than Hofstede's index, this index may also be out of date because it was developed 2 decades ago. We thus call for newer US state level or even county level measures of collectivism in future research. Second, we do not have data at the individual level. It is thus difficult to extrapolate our findings to the individual level. If we had personal level measures of collectivism and objective mobility data, we could test whether personal level collectivism also predicts reduced mobility and whether regional level collectivism has an incremental predictive effect above and beyond personal level collectivism.

Third, the data in the current study is silent on the underlying mechanisms of cultural influence on guideline compliance. Mediation analysis in previous studies revealed that collectivism led to self-report guideline compliance because collectivist individuals (1) were more concerned about others (10), were more likely to perceive guideline compliance as a normative behavior that was considered as important by others (5, 6), trusted the government more (21), and felt less powerlessness (i.e., the sense of being unable to make a meaningful impact on important issues) (22). Collectivistic orientation has also been linked with higher perceived risk of infection (23), which might be another mediator of cultural influence on guideline compliance because people may incline to reduce outside activities if they worry about being infected. Interestingly, besides individuals' mind and behavior, we should also take governmental response into account. It has been observed that the governments in collectivistic regions responded faster to the COVID-19 pandemic (24), which may interact with factors at the individual level to further promote guideline compliance and contain the spread of the virus.

In addition to guideline compliance, collectivism has also been linked with less panic buying (25) and anxiety (26) but higher conspiracy belief (27) during the pandemic. The link between collectivism and less panic buying and anxiety may share some mechanisms with the link between collectivism and guideline compliance, such as higher trust in the government. However, the link between collectivism and conspiracy belief is more complex because conspiracy belief is often found to lower people's intention to engage in preventive behaviors (28). Interestingly, a recent study found conspiracy belief was positively related to preventive actions in South Korea, a typical collectivist country (29). Therefore, the impact of conspiracy belief on preventive behavior may be different or even opposite in individualistic and collectivist regions. We hope future research would examine this issue with more fine-grained analysis.

Taken together, our findings emphasize the importance of cultural values in face of global crises. Although collectivism and individualism are two poles of a single dimension at the aggregated level such as states or countries, they are often considered as two independent dimensions at the individual level (3). Therefore, it would be useful to promote both collectivist and individualistic values at the individual level, given that collectivism might be more helpful for fighting a crisis together while individualism might be more helpful for other outcomes such as creativity and innovation (30, 31).

## Data availability statement

The datasets presented in this study can be found in online repositories. The names of the repository/repositories and accession number(s) can be found below: https://osf.io/xt5qs/?view_only=e7da6b6f69f24bc7b3a9656d00cebb10.

## Author contributions

JD and SX developed the research idea. JD contributed to data analysis and draft writing. SX provided critical feedback for the original draft. Both authors contributed to the article and approved the submitted version.

## Conflict of interest

The authors declare that the research was conducted in the absence of any commercial or financial relationships that could be construed as a potential conflict of interest.

## Publisher's note

All claims expressed in this article are solely those of the authors and do not necessarily represent those of their affiliated organizations, or those of the publisher, the editors and the reviewers. Any product that may be evaluated in this article, or claim that may be made by its manufacturer, is not guaranteed or endorsed by the publisher.
